# Digital Food Records in Community-Based Interventions: Mixed-Methods Pilot Study

**DOI:** 10.2196/mhealth.9729

**Published:** 2018-07-17

**Authors:** Lauren A Fowler, Leah R Yingling, Alyssa T Brooks, Gwenyth R Wallen, Marlene Peters-Lawrence, Michael McClurkin, Kenneth L Wiley Jr, Valerie M Mitchell, Twanda D Johnson, Kendrick E Curry, Allan A Johnson, Avis P Graham, Lennox A Graham, Tiffany M Powell-Wiley

**Affiliations:** ^1^ Cardiovascular Branch Division of Intramural Research National Heart, Lung, and Blood Institute, National Institutes of Health Bethesda, MD United States; ^2^ Columbian College of Arts and Sciences George Washington University Washington, DC United States; ^3^ Clinical Center National Institutes of Health Bethesda, MD United States; ^4^ Blood Epidemiology and Clinical Therapeutics Branch Division of Blood Diseases and Resources National Heart, Lung, and Blood Institute Bethesda, MD United States; ^5^ Division of Genomic Sciences National Human Genome Research Institute National Institutes of Health Bethesda, MD United States; ^6^ Pennsylvania Ave Baptist Church Washington, DC United States; ^7^ College of Nursing and Allied Sciences Howard University Washington, DC United States

**Keywords:** mHealth, diet, community-based participatory research, qualitative research, focus group, obesity

## Abstract

**Background:**

A pressing need exists to understand and optimize the use of dietary assessment tools that can be used in community-based participatory research (CBPR) interventions. A digital food record, which uses a mobile device to capture the dietary intake through text and photography inputs, is a particularly promising mobile assessment method. However, little is understood about the acceptability and feasibility of digital food records in CBPR and how to best tailor dietary assessment tools to the needs of a community.

**Objective:**

The objective of our study was to evaluate the acceptability and feasibility of digital food records among church-based populations in resource-limited wards of Washington, DC, USA, using a mixed-methods approach.

**Methods:**

This community-based pilot study was conducted as part of the Washington, DC Cardiovascular Health and Needs Assessment. Participants (n=17) received a mobile device (iPod Touch) to photodocument their dietary intake for a 3-day digital food record using a mobile app, FitNinja (Vibrent Health). The acceptability of the digital food record was explored through the thematic analysis of verbatim transcripts from a moderated focus group (n=8). In addition, the feasibility was evaluated by the percentage of participants complying with instructions (ie, capturing both before and after meal photos for at least 2 meals/day for 3 days).

**Results:**

Qualitative themes identified were related to (1) the feasibility and acceptability of the mobile device and app, including issues in recording the dietary information and difficulty with photodocumentation; (2) suggestions for additional support and training experiences; and (3) comparisons with other mobile apps. Overall, the participants accepted the digital food record by demonstrating satisfaction with the tool and intent to continue the use (eg, participants recorded an average of 5.2, SD 7, consecutive days). Furthermore, of the 17 participants, 15 photodocumented at least 1 meal during the study period and 3 fully complied with the digital food record instructions.

**Conclusions:**

This study demonstrated digital food records as an acceptable tool in CBPR and identified contributors and barriers to the feasibility of digital food records for future research. Engaging community members in the implementation of novel assessment methods allows for the tailoring of technology to the needs of the community and optimizing community-based interventions.

**Trial Registration:**

ClinicalTrials.gov NCT01927783; https://www.clinicaltrials.gov/ct2/show/NCT01927783 (Archived by WebCite at http://www.webcitation.org/70WzaFWb6)

## Introduction

Cardiovascular disease (CVD) is the leading cause of death in the United States, and modifiable behaviors can drastically reduce the risk for CVD [1]. Poor diet, in particular, is associated with increased risk for CVD [2] and is especially suboptimal among resource-limited populations [3,4], placing these individuals at amplified risk for CVD. Interventions aimed at improving the dietary intake to reduce risk for CVD in this population could be most effective when designed and implemented in partnership with community stakeholders [5-7]. Although reliable and valid assessment tools are fundamental in measuring an intervention’s efficacy, little is known about how best to tailor dietary assessment tools to the needs of a community. Therefore, a pressing need exists to understand and optimize the use of dietary assessment tools that could be used in community-based participatory research (CBPR) interventions.

Traditional dietary measures, such as food frequency questionnaires, 24-hour dietary recalls, recovery biomarkers of nutrient intake, and observational studies, can be costly to implement, burdensome to participants, limited by recall bias, have reduced ecological validity [8-10], and suboptimal in assessing how dietary intake changes over time—an integral measure in intervention research [11]. Ecological momentary assessments (EMAs) allow for data collection to occur in “real time” in participants’ environment, eliminate error associated with participant recall, and increase the ecological validity; however, participants might still perceive a high time burden associated with this method and unintentionally document inaccurate information [8,9,12].

mHealth technology shows considerable potential as a data collection platform. A digital food record (DFR), which involves the use of a mobile device to capture dietary intake through descriptive text and before and after meal photography, is a particularly promising mHealth assessment method that can address some of the limitations of traditional dietary measures and other EMA methods [11-16]. In addition, DFRs have the potential to maximize the validity of the dietary intake data collected by providing researchers with pictures documenting portion sizes consumed, additional undocumented food items, and the rate of food consumption. Moreover, they may also reduce the burden on participants to record the precise intake information [14-17], which could be particularly useful for populations with lower literacy [18,19]. Previous research has demonstrated the feasibility [14,20-23] and validity of DFRs [12,18-20,24-26]. Furthermore, the use of DFRs was shown to have high acceptability and validity in pediatric and adolescent populations [18,19,23,27], a college population [26], overweight and diabetic populations [22,27,28], and a free-living adult population [29].

While numerous studies support the use of DFRs for dietary intake assessment within research contexts [19-21], little is understood about how acceptable and feasible DFRs are for use in CBPR. In fact, the majority of research examining the feasibility of DFRs has been conducted in a laboratory or a controlled setting (eg, provided with meals and snacks to consume at home) [12], suggesting a need to examine the use of DFRs within a community setting. Furthermore, it remains unknown whether DFRs are well suited for resource-limited communities, most of which are at risk for CVD. CBPR provides feedback from community members, captures input on the feasibility of novel tools for use in future studies and interventions, and allows for the “tailoring” of assessment methods to the needs of the community through this involvement [7]. Therefore, using CBPR to examine the usability of DFRs might be most advantageous.

Recent work from our research team assessed the feasibility of a Web-based and wearable technology to measure physical activity among faith-based communities in Washington, DC, USA, where the CVD risk is the highest and resources for physical activity and healthy nutrition are most limited, compared with other wards in DC (NCT01927783 [30]). To complement this work, the feasibility of mHealth technology measurements of the dietary intake were examined within these same communities. Increasing knowledge regarding the usability of digital technology to measure the dietary intake in CBPR could provide opportunities to tailor methods to the needs of the community, improve interventions that promote healthy eating, reduce cardiovascular (CV) health disparities, and improve health outcomes among resource-limited, at-risk populations.

This analysis had two specific aims: (1) to determine the feasibility and acceptability of using a mobile DFR app with a camera to take photographs of dietary intake in an economically disadvantaged population and (2) to examine benefits and barriers to use of DFRs in community-based interventions using a mixed-methods approach. We hypothesized that the use of DFRs is a feasible and acceptable method for capturing the dietary intake in the faith-based community of interest.

## Methods

### Study Design

**Figure 1:**
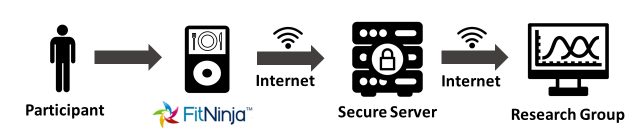
Secure data collection process.

We conducted a CBPR mixed-methods study that incorporated a focus group and pilot testing of a DFR app, FitNinja (Vibrent Health, Fairfax, VA, USA). The data collection process (Figure 1) involved a secure internet server to allow for the secure transfer and uploading of data from the DFR app. To consult on the planning and implementation of this and other community-based initiatives, we established the DC CV Health and Obesity Collaborative (DC CHOC), a community advisory board comprising a diverse group of community representatives and a multidisciplinary research team. Representatives included epidemiologists, behavioral scientists, and community leaders from faith-based organizations, academia, health care, and governmental organizations, as described previously [30]. The first research project designed by the DC CHOC, the Washington, DC CV Health and Needs Assessment, included a subset of studies designed to examine the proposed mobile technology in a sample from the target population before testing on a larger population in the CV Health and Needs Assessment. This series of focus groups and pilot tests were called the CV Health and Needs Assessment Qualitative Study and was the focus of this study. The National Heart, Lung, and Blood Institute Division of Intramural Research Institutional Review Board (Protocol 13-H-0183) approved the CV Health and Needs Assessment and the CV Health and Needs Assessment Qualitative Study. All participants provided written informed consent.

All participants (n=17) received a mobile device (iPod, Apple, Cupertino, CA, USA) and were instructed to take pictures of their meals for a 3-day DFR using the FitNinja mobile app. Participants were instructed to take pictures before and after each meal for at least 3 days (2 weekdays and 1 weekend day) using the mobile app. Participants classified their own meals, with the options of breakfast, lunch, dinner, or a snack. In addition, participants were instructed to take date- and time-stamped photos. Each participant was given a fiducial marker (a 4 × 4-cm card) to place by their meal when taking pictures as a reference image in determining the portion size. While photos were not translated into nutritional or caloric composition data, these were used for descriptive purposes for meals or snacks eaten. Providing descriptive information regarding the meal quality is of unique value for diet counseling focused on the portion size, meal timing, and adherence to “MyPlate” recommendations and can therefore inform other uses of this type of tool.

We conducted a focus group for a subset of qualitative study participants (n=8) after 2 weeks of using the mobile device and FitNinja app. The outcomes of interest in this study were as follows: (1) the feasibility of DFR as measured by the text and photography input in the FitNinja app and (2) the acceptability of the system determined by the results of a moderated focus group discussion. The focus group discussion was designed to elicit participants’ opinions about their experiences with the technology and FitNinja app and to prompt their suggestions for incorporating similar technologies in future behavioral interventions within their communities.

### Digital Food Record

**Figure 2:**
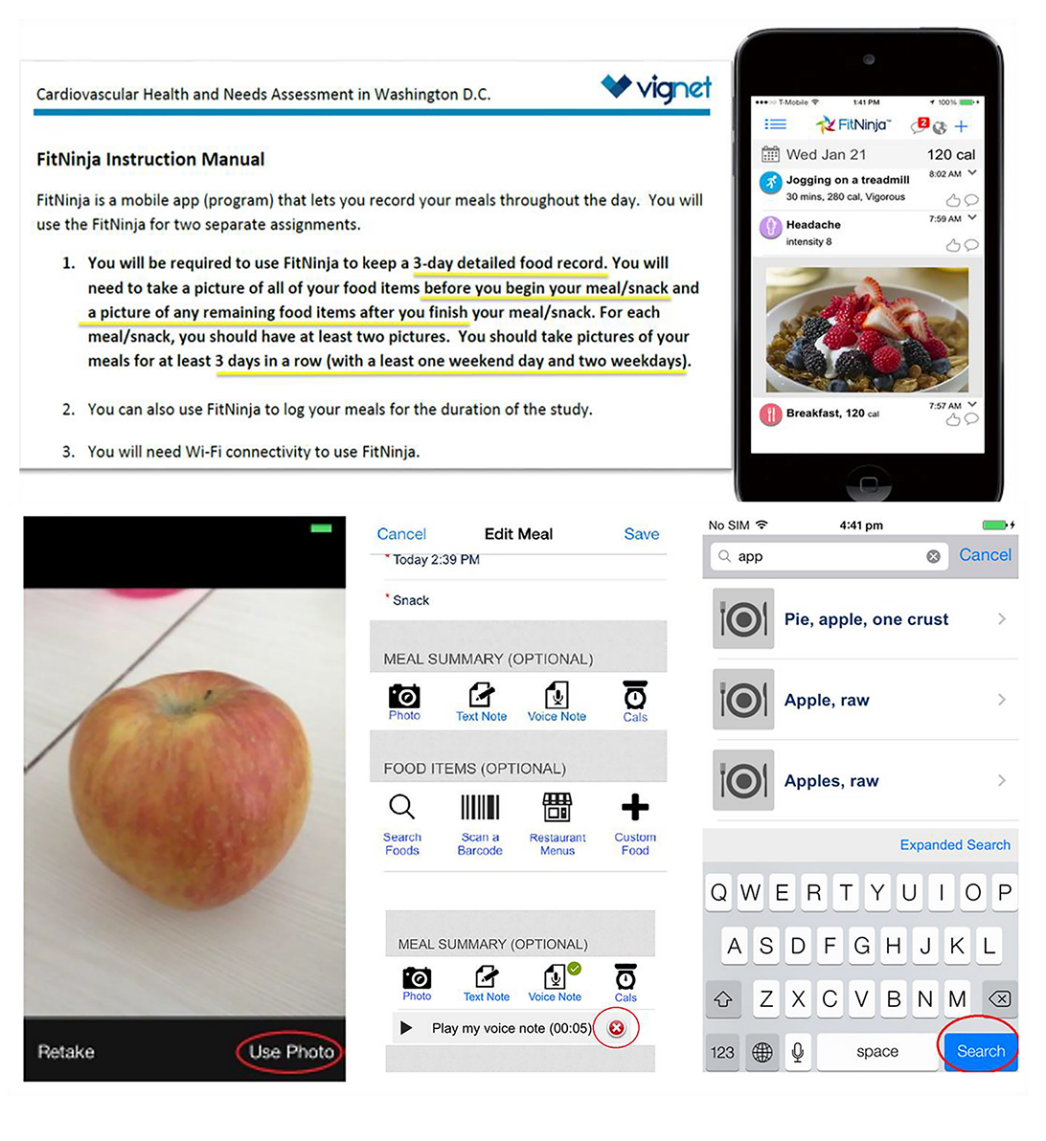
Excerpts from the instruction manual for FitNinja.

Participants were introduced to the FitNinja mobile app and trained on how to use it with a PowerPoint presentation developed by the app developers, with the approval of the research team that paralleled the FitNinja instruction manual. In addition, the training session involved hands-on practice using the app with the support of a research team member. We provided each participant with his or her own instruction manual for reference; the instruction manual provided detailed steps on how to use the app, including how to connect to Wi-Fi, log in to the FitNinja app, take photos of foods consumed, create text and voice notes describing foods consumed, and search the food database. Moreover, the manual described how to scan barcodes of food items, browse restaurant menus, create food items or recipes, add calories, and edit or delete previously recorded meals. Useful tips and clarifications regarding the use of the app were also included as part of the instruction manual, referred to in the manual as “helpful hints.” Figure 2 displays text excerpts from the manual that guided participants through an example of meal recording with photography using the FitNinja app.

The iPod Touch devices were collected from participants after completing up to 30 days of participation. In addition, the data from the devices were uploaded directly to a secured website, where the research team could access and download the data for analysis.

### Focus Group

At the end of the study period, we conducted a focus group for a random subset of participants to provide feedback on their experiences with DFR. Participants in this study were compensated with a US $25 gift card, compatible with the time required for the focus group. The detailed description of the focus group data collection process, including the moderator’s guide that included preselected questions and probes, is available elsewhere [30].

### Study Population

We recruited participants for the CV Health and Needs Assessment Study and its accompanying CV Health and Needs Assessment Qualitative Study from 3 churches in economically disadvantaged wards of Washington, DC, USA,—wards in which the median income was lower than the median income in other wards of DC—where resources for healthy nutritional options were mostly limited and the obesity prevalence was the highest between December 2013 and January 2015 [31,32]. Participants were eligible for this study if they were between the ages of 19 and 85 years and possessed sufficient English language proficiency to execute study tasks. We recruited 8 participants for the CV Health and Needs Assessment Qualitative Study focus group, which was within the recommended range for the qualitative research group discussion of 6-10 participants and comparable with other mHealth initial pilot testing groups [33-36]. Furthermore, additional 9 participants were randomly selected from the larger CV Health and Needs Assessment Study to complete DFR for 17 participants.

### Quantitative Data Analysis

The number of photos captured before and after eating for the 3-day food record was determined for participants to evaluate the feasibility of this tool for the population. For additional quantitative data in the study, we measured food record quality and adherence to food record instructions, including complete adherence to instructions to log “before” and “after” pictures of all recorded meals for 3 consecutive days.

### Qualitative Data Analysis

The focus group was audiorecorded, and the recording was transcribed verbatim by an independent clinical research organization (Social Solutions International, Inc, Silver Spring, MD, USA). The discussion of reliability checks of the transcribing process, development of a codebook of themes, the coding process, and evaluation of the trustworthiness of qualitative analyses are available in previously published work [30,37].

## Results

### Demographic Characteristics

In this study, 53% (9/17) of participants were females, with ages ranging from 28 to 80 years and with an average age of 56.3 (SD 11.8) years. All participants were African American, and the majority was married (n=11), had received at least some postsecondary education (n=14), and reported annual household incomes of more than US $60,000 (n=11), as shown in Table 1.

### Quantitative Study Results

The participation rate for this study was approximately 88%, with 15 of the 17 participants capturing a photo of at least one meal over the study period. We recorded an average of 6 (SD 7.3) days per participant, with an average of 18.2 (SD 23.8) meals (ie, breakfast, lunch, dinner, or snack) recorded over the study period per participant. In addition, consecutive daily recording, defined as the number of days in a row where at least one meal was photodocumented, ranged from 0 (n=2) to 25 days (n=1), with an average of 5.2 (SD 6.6) consecutively recorded days.

**Table 1:** Participants’ characteristics (n=17); CV Health and Needs Assessment Qualitative Study.

Variable		Value
**Sex, n** **(%)**		
	Female	9 (53)
	Male	8 (47)
Age, mean (SD), range		56.3 (12), 28-80
Race (Black or African American), n (%)		17 (100)
**Marital status, n** **(%)**		
	Married	11 (65)
	Single	3 (18)
	Divorced	2 (12)
	Widowed	1 (6)
**Education, n** **(%)**		
	High school diploma or GED^a^	3 (18)
	Some college or technical degree	5 (29)
	College degree	3 (18)
	Graduate or professional degree	6 (35)
Annual household income (>US $60,000), n (%)^b^		11 (69)
Participation rate (logged at least one meal), n (%)		15 (88)
Days recorded, mean (SD)		6.0 (7)
Meals recorded, mean (SD)		18.2 (24)
Consecutive days recorded, mean (SD), range		5.2 (7), 0-25

^a^GED: General Equivalency Diploma.

^b^One participant refused to answer.

**Table 2:** Participation descriptive statistics (n=17); CV Health and Needs Assessment Qualitative Study.

Description	Mean (SD)	Min	Max
Photos per day recorded	2.9 (2.0)	0	10
Photos per meal recorded	1.4 (1.0)	0	9
Meals per day	2.6 (1.2)	0	4
Breakfasts per day	0.8 (0.3)	0	1
Lunches per day	0.6 (0.4)	0	1
Dinners per day	0.7 (0.4)	0	2
Snacks per day	0.5 (0.5)	0	3

**Table 3:** Measures of adherence to the digital food record instructions; CV Health and Needs Assessment Qualitative Study.

Measure of Adherence			Value
**General Adherence, %**			
	Meals with “before” and “after” picture	26.7	
	Meals recorded without pictures	10.6	
	Meals with only “before” picture	57.6	
	Meals with only “after” picture	5.1	
	Percent of days with 3 meals (breakfast, lunch, dinner) per day	39.2	
**Participant Adherence (n=17), n (%)**			
	Participants who logged at least 1 day with ≥3 meals	11 (65)	
	Participants who logged at least 1 meal with a “before” and “after” picture	14 (82)	
	Participants who logged 3 consecutive days with “before” and “after” picture	3 (18)	

The majority of participants recorded their dietary intakes on Sunday, Monday, and Tuesday, with Monday being the modal day in terms of participation rates. The participants logged an average of 2.6 (SD 1.2) meals per day. Breakfast was logged more frequently than other meals, and snacks were logged the least. On average, 2.9 (SD 2.0) photos of meals were taken per day, with an average of 1.4 (SD 1.0) photos taken per meal. Table 2 provides additional descriptive statistics related to DFR.

Most meals (89%) were recorded with one or more photos. However, it was unlikely for participants to log meals with both “before” and “after” photos, with 26.7% of meals having both “before” and “after” pictures recorded. Finally, 18% (3/17) of participants completely adhered to the study directives, which were to record “before” and “after” photos for all meals for 3 consecutive days (Table 3).

Participants included a description with photos about 47% of the time, and the food search feature was used to accompany approximately 16% of the photo records. A common issue with the photos involved was not being able to distinguish all food items in a photo. On average, 67% of the photos had completely distinguishable contents, as determined by a member of the research team (LRY). In determining whether the contents of the photo were distinguishable, the research team member focused on whether all the food items in an image could be recognized (ie, Was the image clear or blurry? Was the photo close enough? Was the whole plate seen? Were all described foods included if a caption was provided? Were the components of a beverage or mixed food item, such as a sandwich, clear?), with the objective of quantifying the feasibility of food photography and not on the validity of food contents. Some of the most common errors in taking photos of meals included being unable to determine what kind of beverage was in a cup or how much of the beverage was consumed; being unable to distinguish the general contents of photos without referring to a text description (if provided); condiments, sauces, or other ingredients were not logged or distinguishable; and portion sizes were indeterminable from photos. For example, condiments and beverages were properly documented 34% and 50% of the time, respectively.

### Qualitative Study Results

#### Themes

The thematic analysis guided the interpretation of the qualitative data. Themes identified within the data collected from the focus group included (1) the iPod or Dietary app, containing 6 related subthemes; (2) support and training; (3) the Hawthorne effect; and (4) device comparisons. Table 4 provides illustrative quotes extracted from the focus group transcript for each subtheme.

**Table 4:** Focus group themes, subthemes, and illustrative quotes; CV Health and Needs Assessment Qualitative Study.

Focus Group Themes		Illustrative Quotes
**1. iPod or** **Dietary App**		
	Feasibility or Acceptability	*For me, I couldn’t do it during the work week…It would be a challenge.* [Male, 55 years] *I had absolutely no problem in taking pictures. I’m retired and whenever I got up and got together I’d take a picture.* [Male, 69 years] *The difference will come in if I take this pictures and sit down with [the researcher], and she’s able to say to me, “On Monday you had bacon, eggs, grits, juice, fruit, and then I see over here you had a sticky bun. Okay, why don’t we cut the sticky bun in half. Eat everything else.”* [Female, 59 years]
	Suggestions for improvement	*I thought it would be cool to have data [on] how I prepared that food…Did I use canola oil, did I use peanut oil? …Did I boil it or did I steam it? Did I put it in the microwave? Did I bake it or did I fry it?* [Male, 55 years]
	Social media	*I would have liked to post this to social media, you know the pictures and calories and everything.* [Male, 49 years]
	Ambiguity over project goals	*Can I ask what the purpose was of wanting to see an empty plate or to see what I decided to leave on the plate?* [Female, 59 years] *I’m glad that I misunderstood [the project goals] so that it energized us [to make our diet healthier] and now we’re off and running.* [Female, 59 years]
	Issues in recording dietary information	*I would take the camera out when eating and put it beside my food so it’s right there to take the picture. But somehow after you finish [eating] you just move on.* [Male, 55 years] *Calorie counting was off…I figured out even though I was scanning the barcodes…I’d look at the calories on my bottles and I looked at the calories on what was scanned and it didn’t match.* [Male, 49 years]
	Feedback	*If there was somebody out there checking in on me…and I could get an email saying great job or here’s something I’d like for you to do…Something like that out there would be a motivator.* [Female, 59 years] *[It would be helpful if someone provided feedback] every 2 weeks, just to check in because you’d hate to go for three weeks or a month and be like we are way off track. You know, so you can make those adjustments as quick as you can.* [Female, 59 years]
**2. Support and Training**		*The helpful hints were very, very helpful.* [Female, 62 years]
	Level of technology literacy	*I think maybe when you’re dealing with computers and people who may be 65, 70, and above you may want to give more instructions on the use of computers.* [Male, 69 years]
**3. Hawthorne Effect**		*I adjusted our diet, bought things that were healthy…fixed things different, presented them differently so that the representation of us would be one that I would be okay with everyone knowing.* [Female, 59 years] *I gave you something [pictures] I thought you might want.* [Female, 59 years]
**4. Device Comparisons**		*My Fitness Pal has also added other devices that it syncs with, other apps it syncs with.* [Female, 43 years] *The one feature that I really like about the Nike trainer is that you get your data…You can see everything instantly on the screen so if I want to share it or track it I have it there instantly when I finish.* [Female, 43 years]

#### Acceptability

The acceptability in feasibility studies can be defined as the extent to which a new measure is judged as suitable, satisfying, or attractive to program recipients [38], and can be evaluated by examining participants’ satisfaction, perceived appropriateness, and intent to continue use, among other outcomes of interest. Several participants conveyed an interest in receiving feedback regarding the quality of their dietary intake, including tips for modifications and behavioral changes, indicating a potential interest in continuing the use of DFR. In addition, some communicated their interest in sharing their dietary record information across social media platforms, suggesting the app would be improved with the addition of a connection to email or other sharing platforms. Overall, the focus group participants showed a strong interest in using the app, recording accurate data, sharing content, receiving feedback, and self-monitoring their behavior and indicated satisfaction with the tool.

#### iPod or Dietary App

Participants acknowledged the extra time burden of completing the food record. One participant stated that “it would be a challenge” to complete the log during the work week. Another acknowledged that retirement had provided them with surplus leisure time, so he had “no problem” photodocumenting his dietary intake.

Feedback on the composition of their diets was a popular suggestion from participants; however, suggestions for the delivery of this feedback varied. One participant stated she would like real-time, immediate feedback from experts by email with suggestions for diet alterations, whereas another suggested an in-person check-in every couple of weeks, “because you’d hate to go for 3 weeks or a month and be like we are way off track…” [Female, 59 years]. Other suggestions for improvement included ideas to increase the depth of data from the records, such as requesting information on food preparation.

Issues associated with recording intake were categorized into two main categories: issues with the iPod or app and issues with consistent photodocumentation. Diligent recorders noticed instances when the number of calories on the nutritional label of a product was incongruent with the number of calories recorded in FitNinja when using the barcode scanner. One participant highlighted that he often had to adjust the serving size from the default serving size after scanning a food item, and this helped to eliminate this discrepancy. In addition, also it was noted that recording was not feasible if Wi-Fi was not available for participants. Furthermore, frustrations were expressed toward the iPod and malfunctions related to the device.

Participants shared their issues with the photodocumentation part of the record, particularly emphasizing the difficulty in remembering to take the “after” picture. One participant said he would place the camera right next to his meal as a reminder to take the “after” picture, but also said that, “somehow after you finish, you just move on.” Another had a rationale for her behavior, stating, “I didn’t always take the after picture. I tried to. Figured since I’m eating everything anyways…” [Female, 59 years]. One of the 2 participants who failed to record any dietary data stated that she failed to record anything because she disliked having to carry two devices (eg, her phone and the iPod for the study); the other participant stated that he did not have adequate time to document his diet.

Several participants shared thoughts about the app that reflected uncertainty in the goals of the study and the purpose of the photodocumentation. One participant stated explicitly that she misunderstood the study goals but was glad she did because it was motivation to improve her and her partner’s diet. Likewise, several other participants echoed this sentiment, agreeing that they interpreted the study as being a behavioral intervention.

#### Support and Training

Two participants in this study shared their frustrations with the technological literacy level necessary to effectively utilize the FitNinja program and the iPod device. Citing their ages, they suggested increased training on more basic functions of device operation, as well as additional training with the app. Favorable comments were made regarding the “helpful hints” provided to participants.

#### Hawthorne Effect

The Hawthorne effect refers to the impact of the evaluation and monitoring without intervention on research participants’ behavior [39,40]. Many participants were acutely aware of the effect of photodocumentation of their diets on their actual behaviors. Participants shared their nutritional improvements with pride and were highly satisfied with this positive effect of DFR on the quality of their diet. Notably, however, 1 participant stated that she would document more of her preparticipation diet, “if I know that there is somebody there to help me adjust.”

#### Device Comparisons

Several focus group participants described the experience using different apps, devices, and programs for self-monitoring their dietary intakes and provided unsolicited comparisons across these devices. They primarily drew attention to features of other technologies that the FitNinja lacked, such as sharing or syncing abilities with other programs and devices (eg, sharing to social media or connecting with a physical activity tracker).

## Discussion

### Principal Findings

Using a mixed-methods approach, this study examined the usability of DFRs among resource-limited communities in Washington, DC, USA. The participation rates were fair; 15 of the 17 total participants recorded at least one meal over the study period, but only 18% (3/17) were totally compliant with instructions to record “before” and “after” photos for all meals for 3 consecutive days. We identified themes through thematic analysis of the focus group transcript related to the feasibility and acceptability, issues in recording, and support and training experiences. The higher likelihood of capturing only the “before” picture of a meal was acknowledged among participants in the focus group. Overall, participants were accepting our photodocumentation tool, as demonstrated by their expressed satisfaction, interest in continued use of the tool, and perceived appropriateness of the measure [38], as well as enthusiastically contributed suggestions for improvements in the DFR app.

### Benefits of Digital Food Record

The benefits of digital recording of dietary intake with inclusion of photography of meals have been well demonstrated across clinical and lab settings [19-21], and its value as a valid assessment tool has been shown previously [24-26]. This pilot study demonstrates that those benefits extend to the use of DFRs in resource-limited community settings. Photodocumentation creates a unique opportunity for researchers to capture patterns of eating, support or enrich textual food records, examine portion sizes, decrease participant burden [19], and potentially improve the validity of dietary assessments [18,19]. The overall acceptability and enthusiasm toward DFR expressed by participants in the focus group demonstrates its potential for use among this population and is similar to previous findings where participants tended to express satisfaction with DFRs [19,26,29]. Photodocumentation is less intensive and burdensome than completing 24-hour dietary recalls or completing daily diet diaries [26] and can be particularly accessible for those with lower levels of literacy [17-19].

Our DFR tool collected data in real-time, providing an opportunity to provide real-time feedback to community members. Participants in the focus group were open and eager to receive feedback regarding their diets.. Previous work has examined the effect of daily feedback messages on changes in diet among obese adults, finding favorable effects [41]. While many participants expressed interest in receiving feedback, the preferred delivery method (eg, in-person vs electronically) and preferred time frame (eg, real-time vs twice a month) varied across participants. Interventions with DFRs might consider designing feedback options that can be tailored according to participants’ preferences or determining which method and dosage are optimal for the behavioral change. Overall, this study suggests that this tool would be a well-accepted delivery method for dietary interventions among community populations.

### Barriers to the Use of Digital Food Records in Community Settings

As far as can be determined, this feasibility pilot study is the first to use qualitative data to identify barriers to the use of DFRs within a community setting. One prominent theme within the qualitative data was technology literacy and prior device knowledge. While participants were trained to use the app, they likely began the study with varying levels of experience with technology. Of note, 2 participants cited their age stating that they had difficulty in using the technology, and 1 mentioned he had his daughter demonstrate how to operate some features of the device and app. It may be useful for future studies using DFRs to measure eHealth literacy [42] and include advanced training sessions for those participants who might have lower technology literacy or request additional training.

Participants expressed difficulty in remembering to take “before” and “after” meal pictures, particularly with taking “after” meal pictures. The willingness of participants to devise strategies to improve their documentation and the photodocumentation rates of “after” meal photos suggests that participants might benefit from a tailored system of before or after meal reminders. Previous studies have addressed this barrier by sending either standard or customized EMA prompts to participants’ mobile phones reminding them to photodocument [24] or by eliminating the burden of photodocumentation with wearable, automated cameras [15,43]. However, more research is warranted to completely understand issues involved with wearable cameras for capturing the food intake, especially in community settings. Another potential strategy to improve photodocumentation rates would involve greater articulation of study goals by the research team during participant training. Some participants expressed uncertainty about the purpose of the photodocumentation part of the food record. It is possible that the utility of photos was underemphasized, thereby resulting in less documentation. It is also possible that the participants might not have taken the after picture because they did not understand the point of taking a picture of an empty plate. In this case, the inclusion of a checkbox that can be used to indicate that the entire meal was consumed could be beneficial for both participants and researchers.

This pilot study revealed that the quality of images captured by participants could be improved. It might be necessary to introduce photo quality control methods to the app, such as those proposed by the Remote Food Photography Method [24], whereby computer software surveys image data to detect missing or poor quality imagery and sends tailored prompts to participants to improve the overall data quality. In addition, the software could require participants to retake the poor quality photograph to enable all contents to be distinguishable, and food recognition software could identify and estimate the energy and nutrient content of any other detected food items in the image [44].

In addition, participants might have changed their eating behavior merely because of participating in this study and monitoring and recording their food intake, as described by the Hawthorne effect. Diet self-monitoring is a common theoretically based behavioral change strategy [45], and a review of self-monitoring in weight loss research found consistent positive effects of adherence to diet self-monitoring [46], particularly when using mobile technology [40]. Hawthorne effects might not have been all positive in this study. For example, the purposeful omission of certain meals, snacks, or drinks that are associated with socially undesirable outcomes is possible. This is, however, a weakness of all self-reported dietary measures. Although Hawthorne effects could have contributed to errors in reporting or changes in the eating behavior of participants, they do not affect the conclusions of this study. A rigorous study design will prevent Hawthorne effects from weakening tests of intervention efficacy and effectiveness with future use of DFRs. In fact, the self-monitoring aspect of recording data in real-time inherent in DFRs enhances their value in intervention research by allowing them to serve as both an assessment tool and a behavioral change strategy [47].

### Limitations

Participation in DFR was extremely variable. Indeed, the data collected show that 2 participants failed to take *any* photographs over the study period. While this could be from participants’ nonresponse, it could also be a result of technological issues (eg, participants had issues with the device). However, gathering qualitative data allowed us to examine rich accounts of participants’ experiences with DFR and to hypothesize strategies to decrease participants’ nonresponse. In addition, this study allowed us to examine participation rates outside of a laboratory setting in the participants’ natural environments, where life pressures can detract from consistent recording. Furthermore, this study is limited by its inclusion of one focus group, and future studies should include multiple focus groups.

This study did not examine the validity of the food records. Future studies should involve larger sample sizes and test the psychometric properties of DFRs to examine the reliability and validity of these dietary assessment methods within CBPR, as well as compare DFRs to a standard measure of dietary intake, such as a 24-hour diet recall [48] or objectively measured energy expenditure (doubly labeled water) [12,14,20]. Furthermore, conclusions regarding the longer-term use of DFR cannot be drawn. Future work should use a longer study period to examine engagement, adherence, attrition, and retention.

Finally, the FitNinja app was available for participants to use and log their food intake over Wi-Fi. Notably, data collection is not feasible in Wi-Fi-limited regions. Reliance on Wi-Fi for documentation may, therefore, not be ideal in some populations.

### Conclusions and Recommendations

To the best of our knowledge, this study is one of the first to demonstrate the feasibility and acceptability of DFRs in a faith-based community setting. The participation rates were fair, leaving room for improvement. This study identified many barriers to the use of digital food tracking tools (eg, inconsistent use and indistinguishable pictures), providing opportunities for future research to address these barriers. For instance, it might be useful for future studies to develop, implement, and test the efficacy of techniques to increase the adherence to DFR instructions, such as implementing a tailored system of reminders to increase before or after meal photodocumentation. In addition, future work could aim to decrease ambiguity and increase motivation or participation by improving the communication of project goals and the utility of digital records. To address technological and app literacy issues, follow-up studies using DFR might wish to provide several community participants with additional, extensive training and designate these “super-users” as expert resources within the community who can provide assistance to other users if issues or questions arise.. Furthermore, future interventions could use our DFR to deliver timely feedback to individuals about their diet. Finally, higher consideration should be given to identifying potential barriers to the use of digital food tracking apps across diverse populations, particularly if these tools are to be suggested for use in interventions or clinical settings to promote improvement in diet.

Community-based and engaged research allows for the tailoring of technology to the needs of the community. Therefore, it is essential to gain community feedback when testing novel assessment methods and to address barriers to their use. Until health equity is achieved, researchers must examine the use of novel technologies among at-risk and vulnerable populations, where interventions are most vital. Increasing the knowledge regarding the usability of digital technology to measure dietary intake in CBPR could improve interventions that promote healthy eating and reduce CV health disparities.

## Abbreviations

CBPR: community-based participatory research
CVD: cardiovascular disease
DFR: digital food record
EMA: ecological momentary assessments
